# A phase I study of the investigational NEDD8-activating enzyme inhibitor pevonedistat (TAK-924/MLN4924) in patients with metastatic melanoma

**DOI:** 10.1007/s10637-016-0348-5

**Published:** 2016-04-08

**Authors:** Shailender Bhatia, Anna C. Pavlick, Peter Boasberg, John A. Thompson, George Mulligan, Michael D. Pickard, Hélène Faessel, Bruce J. Dezube, Omid Hamid

**Affiliations:** Department of Medicine/Medical Oncology, University of Washington Medical Center/Fred Hutchinson Cancer Research Center/Seattle Cancer Care Alliance, 825 Eastlake Ave W, G4-830, Seattle, WA 98109-1023 USA; Departments of Medicine (Perlmutter Cancer Center) and Dermatology, NYU Langone Medical Center, New York, NY USA; The Angeles Clinic and Research Institute, Translational Research & Cutaneous Oncology, Los Angeles, CA USA; Millennium Pharmaceuticals, Inc., a wholly owned subsidiary of Takeda Pharmaceutical Company Limited, Cambridge, MA USA

**Keywords:** Melanoma, NAE inhibition, Nrf-2, Phase I, Pevonedistat

## Abstract

*Purpose* The therapeutic index of proteasome inhibitors may be improved through selective inhibition of a sub-component of the ubiquitin-proteasome system, such as the NEDD8-conjugation pathway. This multicenter, phase I, dose-escalation study assessed safety and the maximum tolerated dose (MTD), pharmacokinetics, pharmacodynamics, and antitumor activity of pevonedistat, an investigational NEDD8-activating enzyme (NAE) inhibitor, in patients with metastatic melanoma. *Methods* Patients received intravenous pevonedistat on Days 1, 4, 8, 11 (schedule A) or 1, 8, 15 (schedule B) of 21-day cycles. *Results* 26 patients received pevonedistat 50–278 mg/m^2^ on schedule A; 11 patients received pevonedistat 157 mg/m^2^ on schedule B. The schedule A MTD was 209 mg/m^2^: dose-limiting toxicities (DLTs) included grade 3 hypophosphatemia and grade 3 increased blood creatinine (associated with grade 3 hyperbilirubinemia). Two schedule A patients experienced acute organ failure toxicities, one of whom experienced grade 5 acute renal failure. Dose escalation did not occur in schedule B: DLTs included grade 3 myocarditis, grade 2 acute renal failure, and grade 2 hyperbilirubinemia in a single patient. Pevonedistat pharmacokinetics were approximately dose-proportional across the dose range studied, with a biphasic disposition profile characterized by a short elimination half-life (~10 h). Pharmacodynamic studies showed increases in NAE-regulated transcripts post-treatment; all post-dose biopsy samples were positive for pevonedistat-NEDD8 adduct. One schedule A patient achieved a partial response; 15 patients had stable disease (4 lasting ≥6.5 months). *Conclusions* Pevonedistat was generally well tolerated at the MTD. Anticipated pharmacodynamic effects of NAE inhibition were observed with single-agent pevonedistat in peripheral blood and tumor tissue.

**Electronic supplementary material:**

The online version of this article (doi:10.1007/s10637-016-0348-5) contains supplementary material, which is available to authorized users.

## Introduction

The ubiquitin–proteasome system (UPS) plays a critical role in regulating intracellular proteins in eukaryotic cells, including key substrate proteins that mediate cell growth and survival, cellular signaling, and transcription factor regulation [1]. Dysregulation of the UPS has been implicated in cancer development and progression [2]. The clinical success of proteasome inhibitors such as bortezomib and carfilzomib has validated the UPS as a rational target for cancer therapy [3]. However, these proteasome inhibitors result in broad inhibition of protein degradation, which accounts for their toxic effects. The therapeutic index of proteasome inhibitors may be improved through selective inhibition of a sub-component of the UPS, such as the neddylation pathway, as described below.

The UPS regulates degradation of intracellular proteins by tagging substrate proteins with a polyubiquitin chain, which marks them for subsequent degradation by the proteasome [4]. The polyubiquitination process is mediated by E3 ligases. Cullin-RING ligases (CRLs), a subgroup of the E3 ligases, are especially important in the degradation of several proteins relevant to oncology, including the cyclin-dependent kinase inhibitor p27, Cdt1 (chromatin licensing and DNA replication factor-1), and Nrf-2 [nuclear factor (erythroid derived 2)-related factor 2] [5]. CRLs are activated via the neddylation pathway [6], which involves conjugation of the ubiquitin-like protein NEDD8 (neural precursor cell expressed, developmentally down-regulated 8) to the CRLs. NEDD8 conjugation is essential for the E3 ligase activity of CRLs [5].

The NEDD8-activating enzyme (NAE) is a critical regulator of the neddylation pathway [6]. Inhibition of NAE can inhibit the activity of the CRLs and result in accumulation of CRL substrate proteins [5, 6]. Pevonedistat (TAK-924/MLN4924) is a first-in-class, investigational, small-molecule inhibitor of NAE [7]. It forms a covalently bound adduct with NEDD8 while bound to NAE [8]. NAE inhibition via pevonedistat prevents the proteasomal degradation of a fraction (~20 %) of the proteins regulated by the UPS, in contrast to the broad inhibition seen with proteasome inhibitors [5–7, 9, 10]. This fraction includes several proteins involved in tumorigenesis, such as the tumor suppressor ICER [11], and proteins involved in the modulation of transcription, cell cycle control, and apoptosis, such as ING3 [12].

There are extensive preclinical data supporting the potential utility of pevonedistat in melanoma. Pevonedistat is cytotoxic in a range of hematologic and solid tumor cell lines [10, 13–18], and has shown antitumor activity in a number of in-vivo models [15, 16, 19, 20]. The mechanism of action primarily involves stabilization of Cdt1 and induction of the DNA damage response [9, 13, 21, 22], but likely also includes autophagy [14, 23] and inhibition of nuclear factor-κB through stabilization of I-κBα [16, 19]. Dysregulation of the UPS has been implicated in the development and progression of melanoma [11, 12]; thus, pevonedistat has been investigated in preclinical studies in melanoma cell lines and tumor xenograft models, and has demonstrated cytotoxicity and antitumor activity [24–28]. Pevonedistat-mediated cell death in melanoma cell lines appears to involve inhibition of cellular phase transition following the induction of DNA re-replication stress [24]. Pevonedistat was associated with tumor growth inhibition and regression in patient-derived melanoma tumor mouse xenograft explant models [27]. Preliminary clinical activity of pevonedistat in melanoma was also noted in the first-in-human phase I study; in nine melanoma patients, one achieved a partial response and another achieved stable disease lasting 6 months [29]. Investigation in melanoma patients also offered the feasibility of repeated skin tumor biopsies to facilitate pharmacodynamic (PD) investigations.

This phase I study (NCT01011530) was conducted to assess the safety, pharmacokinetics (PK), PD, and antitumor activity of pevonedistat in patients with metastatic melanoma. The study tested two administration schedules of pevonedistat. Paired pre-dose and post-dose tumor biopsies were acquired in patients treated at the maximum tolerated dose (MTD) to evaluate the PD effects of NAE inhibition in this clinical setting. The study results have helped inform the overall clinical development of pevonedistat.

## Patients and methods

### Patients

Eligible patients were ≥18 years old, and had a diagnosis of metastatic melanoma, an Eastern Cooperative Oncology Group (ECOG) performance status of 0–2, adequate hematologic, hepatic, renal, and cardiovascular function, and radiographically or clinically evaluable tumor. Patients enrolled in the expansion cohort required measurable disease as defined by the Response Evaluation Criteria in Solid Tumors (RECIST) criteria (v1.1). Patients with brain metastases were eligible if they were asymptomatic and had stable neurologic status for ≥2 weeks after completion of local therapy (surgery or radiation).

Patients were excluded if they had received radiotherapy, systemic antineoplastic therapy, investigational agents, CYP3A inhibitors/inducers, major surgery, serious infection, or antibiotic therapy within 14 days of first dose of pevonedistat. Patients with known HIV or hepatitis B infection or known/suspected active hepatitis C infection were excluded, as were patients with a history of coagulopathy or bleeding disorder. The study was conducted in accordance with the ethical principles originating in or derived from the Declaration of Helsinki and its amendments and in accordance with 21 Code of Federal Regulations 50 / 56 / 312. Institutional review boards at each of the participating investigational centers approved the study. All patients provided written informed consent.

### Study design

This was an open-label, multicenter, phase I dose-escalation study. The primary objectives were to determine the safety profile, establish the MTD, and inform the recommended phase II dose and dosing schedule for pevonedistat. Secondary objectives were to evaluate the PK and antitumor activity of pevonedistat, and to investigate its PD effects in blood and tumor samples.

Patients received pevonedistat as 1 h intravenous infusions either on Days 1, 4, 8, and 11 (schedule A) or on Days 1, 8, and 15 (schedule B, weekly dosing) of 21-day cycles. For schedule A, intermittent dosing on Days 1, 4, 8, and 11 was chosen to match the twice-weekly schedule of the proteasome inhibitor bortezomib [30]. The starting dose of 50 mg/m^2^ was selected based on the toxicities and the MTD observed in another phase I study that investigated pevonedistat administration on Days 1–5 of 21-day cycles in patients with advanced solid tumors. It was hypothesized that intermittent dosing – extending the dose over a 2-week period – would be better tolerated than the continuous dosing schedule, which was associated with severe hepatotoxicity [29]. Schedule B, which was added subsequently through a protocol amendment, was based on the rationale that weekly bortezomib has similar efficacy and is more convenient than the twice-weekly schedule [31, 32]. The starting dose for weekly dosing of pevonedistat on schedule B (157 mg/m^2^) was based on data indicating that this dose was well tolerated on a Day 1, 4, 8, and 11 schedule in a phase I study in multiple myeloma and lymphoma [33]. Patients could receive pevonedistat until disease progression or unacceptable drug-related toxicity, up to a maximum of 12 months.

Dose escalation proceeded via a Bayesian continual reassessment method using 2-patient cohorts and 1.33-fold dose increments over the previous dose level. Dose escalation decisions were based on occurrence of DLTs in Cycle 1 and the predicted MTD from the continual reassessment model (Supplementary Fig. S1). The MTD was defined as the dose level at which 6 patients had been treated and at which the algorithm did not recommend dose de-escalation. Additional patients were to be enrolled at the MTD to further evaluate safety.

DLTs were defined as: grade 4 neutropenia for >7 consecutive days or grade 3 neutropenia with fever (oral temperature ≥38.5 °C) and/or infection; grade 4 thrombocytopenia for >7 consecutive days, platelets <10,000/mm^3^ at any time, or grade 3 thrombocytopenia with bleeding; grade ≥3 nausea or diarrhea despite optimal anti-emetic prophylaxis or supportive therapy, or any other grade ≥3 nonhematologic toxicity (except arthralgia/myalgia, brief [<1 week] fatigue, or fever occurring in the absence of grade ≥3 neutropenia); a decrease in LVEF to <40 % or an absolute reduction of ≥10 % to <50 %; an increase in pulmonary artery systolic pressure, as determined by echocardiogram, to >50 mmHg or three times that at baseline; treatment delay of >1 week due to lack of recovery from drug-related toxicities, or other drug-related toxicity requiring doses to be missed or therapy to be discontinued.

### Assessments

Adverse events (AEs) were graded using the National Cancer Institute’s Common Terminology Criteria for AEs version 3.0. Response was assessed using the modified RECIST guideline (v1.1) [34]. Computed tomography or magnetic resonance imaging scans were performed at screening, at the end of Cycle 2, and then every other cycle.

Blood samples (3 mL) for PK analysis were collected during Cycle 1. On schedule A, serial samples were collected within 1 h before dosing on Day 1, immediately after completion of infusion (immediately before switching off infusion pump), and at 1, 4, and 7 h after completion of infusion. Samples were also collected on Days 2 and 3; on Days 4 and 8 (immediately before dosing and immediately after completion of infusion); on Day 11 immediately before dosing, immediately after completion of infusion, and at 4 and 7 h after completion of infusion; and on Day 15. On schedule B, blood samples were collected within 1 h before dosing on Day 1, immediately after completion of infusion (immediately before switching off infusion pump), and at 1, 2, 4, and 7 h after completion of infusion. Samples were also collected on Days 2, 3, and 4; and on Days 8 and 15 (immediately before dosing and immediately after completion of infusion). Samples were analyzed at Tandem Labs (West Trenton, NJ) for pevonedistat plasma concentrations using Good Laboratory Practice-validated liquid chromatography/tandem mass spectrometry methods. The dynamic ranges were 1–500 ng/mL for the low-range assay and 75–7500 ng/mL for the high-range assay.

Blood samples for PD analysis were collected during Cycle 1. On schedule A samples were collected: at screening; on Days 1 and 11 within 1 h before dosing and at 4 and 7 h after completion of infusion; and on Days 4 and 15. On schedule B samples were collected: at screening; on Day 1 within 1 h before dosing and 1, 2, 4, and 8 h after completion of infusion; on Day 8 within 1 h before dosing; and on Day 15 within 1 h before dosing and 1, 2, and 4 h after completion of infusion. Gene expression of NAE-regulated transcriptional targets (ATF3, GCLM, GSR, MAG1, NQ01, SLC7A11, SRXN1, and TXNRD1) in whole blood was analyzed by reverse-transcription polymerase chain reaction (RT-PCR). Raw data were transformed prior to calculation of percent change and assumptions were made for missing data, as previously described [33]. Summary statistics were generated for the percent change from baseline at each time point for each gene.

For patients in the MTD expansion cohort, paired tumor biopsies were obtained at screening and at 3–6 h post-dosing on Day 4 (schedule A) or Day 8 (schedule B). Immunohistochemistry was used to detect pevonedistat–NEDD8 adduct (to demonstrate penetration of the drug into tumor tissue and the formation of the expected entity upon NAE inhibition), and to determine levels of Cdt1 and Nrf-2 expression (to demonstrate anticipated PD effects arising from NAE inhibition). Immunohistochemical analyses were performed at Millennium Pharmaceuticals, Inc. as previously described [33].

### Statistics

All results were summarized descriptively, and no formal hypothesis testing was conducted. The safety population included all patients who received ≥1 dose of pevonedistat. The DLT-evaluable population comprised patients who received all scheduled doses during Cycle 1 or experienced a DLT during Cycle 1. The PK-evaluable population included all patients who received all protocol-defined doses in Cycle 1, had sufficient plasma concentration–time data to reliably estimate PK parameters, and had not received any excluded concomitant medications per protocol. Individual pevonedistat plasma concentration–time data were analyzed by noncompartmental methods using WinNonlin software (Version 6.2, Pharsight Corporation, Cary, NC). Plasma concentrations below the lower limit of quantification were set to zero for analysis. The response-evaluable population included all patients who received ≥1 dose of pevonedistat, had measurable disease at baseline per RECIST [34], and had ≥1 post-baseline disease assessment.

## Results

### Patients

A total of 37 patients were enrolled and received at least one dose of pevonedistat at one of three sites in the United States from December 2009 to May 2012. Twenty six patients received pevonedistat on schedule A and 11 patients received pevonedistat on schedule B. Patients’ baseline demographics and disease characteristics are summarized in Table 1. Thirty one (84 %) patients had skin melanoma as the primary site. Of the 15 patients whose tumor could be assessed for *BRAF* mutation status, 10 (67 %) patients had wild-type *BRAF*, and 5 (33 %) patients had a *BRAF V600E* mutation.

**Table 1:** Baseline patient demographics and disease characteristics

Characteristic	Schedule A (*n* = 26)	Schedule B (*n* = 11)	Total (*N* = 37)
Median age, years (range)	62.9 (34–79)	57.8 (33–76)	61.8 (33–79)
Male, *n* (%)	16 (62)	7 (64)	23 (62)
Race, *n* (%)			
White	26 (100)	10 (91)	36 (97)
Asian	0	1 (9)	1 (3)
ECOG performance status, *n* (%)			
0	13 (50)	2 (18)	15 (41)
1	13 (50)	9 (82)	22 (59)
Primary site, *n* (%)			
Melanoma of the skin	22 (85)	9 (82)	31 (84)
Other melanoma*	4 (15)	2 (18)	6 (16)
Disease Stage, *n* (%)			
III (unresectable)	5 (19)	3 (27)	8 (22)
IV	16 (62)	8 (73)	24 (65)
Not available	5 (19)	0	5 (14)
LDH > ULN, *n* (%)^†^	12 (48)	7 (70)	19 (54)
>2 x ULN, *n* (%)	3 (12)	3 (30)	6 (17)
Prior therapy, *n* (%)			
Prior antineoplastic therapy	25 (96)	11 (100)	36 (97)
Prior radiation	18 (69)	10 (91)	28 (76)
Prior surgery or non-radiation procedure	25 (96)	9 (82)	34 (92)

*M1c melanoma ocular, malignant melanoma of the conjunctiva, malignant melanoma of the uvea, melanoma – left ear, nasal melanoma, ocular choroidal melanoma, each *n* = 1. ^†^ *N* = 35; LDH data not available at baseline in 2 patients, 1 in each schedule

*ECOG* Eastern Cooperative Oncology Group, *LDH* lactate dehydrogenase, *ULN* upper limit of normal

All patients had discontinued pevonedistat at the time of data cut-off. On schedule A, 21 (81 %) patients came off study upon experiencing progressive disease (*n* = 20) or symptomatic deterioration (*n* = 1); the remaining 5 (19 %) patients discontinued treatment due to AEs (*n* = 2), withdrawal (*n* = 2), and lengthy treatment hold unrelated to study drug (*n* = 1). On schedule B, 8 (73 %) patients came off study upon experiencing progressive disease, and 3 (27 %) patients discontinued due to AEs (*n* = 2) or withdrawal (*n* = 1).

### Dose escalation, DLTs, and MTD determination

On schedule A, 26 patients received pevonedistat at one of 7 different dose levels, including 2 patients each at 50, 67, and 89 mg/m^2^, 5 at 118 mg/m^2^, 2 at 157 mg/m^2^, 11 at 209 mg/m^2^, and 2 at 278 mg/m^2^. A total of 19 patients were DLT-evaluable. No DLTs were reported at the first three dose levels. At the 118 mg/m^2^ dose level, one patient experienced asymptomatic drug-related grade 3 hypophosphatemia on Day 4 of Cycle 1, which resulted in a dose reduction to 89 mg/m^2^; the hypophosphatemia did not recur at the lower dose. A total of 5 patients were enrolled at 118 mg/m^2^ and no other DLTs were recorded. One patient at the 118 mg/m^2^ dose level was hospitalized on Day 3 of Cycle 1 due to grade 3 acute respiratory failure, which resolved on Day 5; this patient resumed pevonedistat at a reduced dose of 89 mg/m^2^ and tolerated it well. Although the investigator did not initially report this as a DLT, attributing it to underlying lung metastases, it was eventually concluded that this event had met the protocol-specified definition of a DLT. Subsequently, no DLTs were reported at the 157 or 209 mg/m^2^ dose levels. At the 278 mg/m^2^ dose level, one patient experienced drug-related grade 3 increased blood creatinine and grade 3 increased blood bilirubin on Day 3 of Cycle 1, which resulted in hospitalization and treatment discontinuation. These events subsequently resolved without long-term sequelae. No further patients were enrolled at 278 mg/m^2^ due to the severity of the DLT at this dose level. Four additional patients were enrolled at the 209 mg/m^2^ dose level. In the absence of any recorded DLTs among the initial 6 patients enrolled at this dose level, the MTD of pevonedistat on schedule A was determined to be 209 mg/m^2^, and another 5 patients were enrolled to further evaluate safety. Among these 5 dose-expansion patients, two experienced AEs that met the definition for DLTs; both patients had dose reductions to 157 mg/m^2^. One patient had drug-related grade 1 increased aspartate aminotransferase, and one patient had drug-related grade 1 increased B-type natriuretic peptide. Additionally, one dose-expansion patient with a history of renal insufficiency was hospitalized on Day 2 of Cycle 1 due to grade 4 acute renal failure and grade 4 acute hepatic failure, and subsequently died on day 9 due to acute renal failure.

On schedule B, all 11 patients received pevonedistat at the 157 mg/m^2^ dose level. Dose escalation did not occur due to the Sponsor’s decision to stop enrollment following a program-wide review. Ten patients were DLT-evaluable. One patient experienced DLTs of grade 3 myocarditis on Day 1, and grade 2 acute renal failure and grade 2 hyperbilirubinemia on Day 2 of Cycle 1, which resulted in hospitalization and treatment discontinuation. The exact cause of these DLTs was unclear, but was thought to be secondary to direct effects of pevonedistat on the kidneys, biliary tree, and myocardium.

### Pevonedistat exposure and safety profile

All 37 patients received at least one dose of pevonedistat and were included in the safety population. The median number of treatment cycles of pevonedistat administered was 2 (range 1–16) for the entire study population, with a median of 4 (range 1–16) on schedule A and a median of 2 (range 1–11) on schedule B. The intensity of pevonedistat dosing, defined as the dose received as a proportion of the dose expected, was ≥80 % in 19 (73 %) patients on schedule A and 8 (73 %) patients on schedule B.

The safety profile of pevonedistat is summarized in Table 2. All patients experienced at least one AE. The most common AEs regardless of causality are shown in Supplementary Table S2 and included fatigue (*n* = 25; 68 %), diarrhea (*n* = 18; 49 %), anemia (*n* = 15; 41 %), myalgia (*n* = 15; 41 %), nausea (*n* = 13; 35 %), constipation (*n* = 12; 32 %), vomiting (*n* = 12; 32 %), arthralgia (*n* = 11; 30 %), decreased appetite (*n* = 11; 30 %), dizziness (*n* = 10; 27 %), and peripheral neuropathy (*n* = 10; 27 %). Peripheral neuropathy was mild (grade 1) in all 10 patients; due to a lack of comprehensive data on prior therapies, we could not address the possibility that prior treatment with neurotoxic agents (such as taxanes, platinum-based therapies, or vinca alkaloids) predisposed these patients to develop neuropathy with pevonedistat. Common drug-related AEs are listed in Table 2. Overall, 18 (49 %) patients experienced grade ≥3 AEs; the only grade ≥3 AEs, regardless of causality, that were reported in more than one patient were anemia (*n* = 5; 14 %), benign/malignant neoplasms, and small intestinal obstruction (each *n* = 2; 5 %) (Supplementary Table S2). Grade ≥3 AEs assessed as drug-related by the investigators are shown in Table 2.

**Table 2:** Safety profile of pevonedistat, including drug-related AEs reported in at least 10 % of patients overall, and all drug-related grade ≥3 AEs

AE, *n* (%)	Schedule A (*n* = 26)	Schedule B (*n* = 11)	Total (*N* = 37)
Any AE	26 (100)	11 (100)	37 (100)
Any drug-related AE	25 (96)	10 (91)	35 (95)
Common drug-related AE (≥10 % of patients):			
Fatigue	12 (46)	6 (55)	18 (49)
Myalgia	10 (38)	4 (36)	14 (38)
Diarrhea	10 (38)	2 (18)	12 (32)
Nausea	8 (31)	3 (27)	11 (30)
Anemia	6 (23)	4 (36)	10 (27)
Peripheral neuropathy	7 (27)	3 (27)	10 (27)
Vomiting	6 (23)	3 (27)	9 (24)
Arthralgia	4 (15)	2 (18)	6 (16)
Decreased appetite	5 (19)	1 (9)	6 (16)
Pyrexia	4 (15)	1 (9)	5 (14)
AST increased	5 (19)	0	5 (14)
GGT increased	5 (19)	0	5 (14)
Blood ALP increased	5 (19)	0	5 (14)
Chills	3 (12)	1 (9)	4 (11)
ALT increased	4 (15)	0	4 (11)
Night sweats	3 (12)	1 (9)	4 (11)
Any grade ≥3 AE	12 (46)	6 (55)	18 (49)
Any drug-related grade ≥3 AE	7 (27)	4 (36)	11 (30)
Drug-related grade ≥3 AEs (>1 patient)*:			
Anemia	1 (4)	1 (9)	2 (5)
Any serious AE	9 (35)	5 (45)	14 (38)
Any drug-related serious AE	5 (19)	1 (9)	6 (16)
AE resulting in study drug discontinuation	2 (8)	2 (18)	4 (11)
On-study death	2 (8)	1 (9)	3 (8)

*The following drug-related grade ≥3 AEs were reported in 1 patient each: acute hepatic failure, acute renal failure, acute respiratory failure, angina pectoris, anuria, arthralgia, blood bilirubin increased, blood creatinine increased, confusional state, dyspnea exertional, fatigue, hepatic encephalopathy, hyponatremia, hypophosphatemia, hypotension, myocarditis, respiratory distress, respiratory failure, and syncope

*AE* adverse events, *ALP* alkaline phosphatase, *ALT* alanine aminotransferase, *AST* aspartate aminotransferase, *GGT* gamma-glutamyltransferase

Overall, 14 (38 %) patients experienced at least one serious AE (SAE), with 6 (16 %) experiencing at least one drug-related SAE. Four (11 %) patients had AEs that resulted in discontinuation: a patient receiving pevonedistat 209 mg/m^2^ on schedule A had drug-related grade 4 acute renal failure; a patient receiving pevonedistat 278 mg/m^2^ on schedule A discontinued due to the DLT of drug-related grade 3 increased blood creatinine and drug-related grade 3 increased blood bilirubin; a patient receiving pevonedistat 157 mg/m^2^ on schedule B discontinued due to grade 3 small intestinal obstruction (associated with multifocal abdominal subcutaneous metastatic deposits), which was considered unrelated to treatment; a second patient on schedule B discontinued due to the DLTs of grade 3 myocarditis, grade 2 acute renal failure, and grade 2 hyperbilirubinemia.

Three patients died on study, within 30 days of their last dose of pevonedistat. One patient treated at 118 mg/m^2^ on schedule A received four doses of pevonedistat, discontinued due to symptomatic deterioration not related to treatment, and died 26 days after the Cycle 1, Day 11 dose. A patient treated at 209 mg/m^2^ on schedule A received one dose of pevonedistat and died due to drug-related acute renal failure on Day 9 of Cycle 1. One patient on schedule B died 30 days after the Cycle 2, Day 15 dose due to progressive disease.

### Pharmacokinetics

**Fig. 1:**
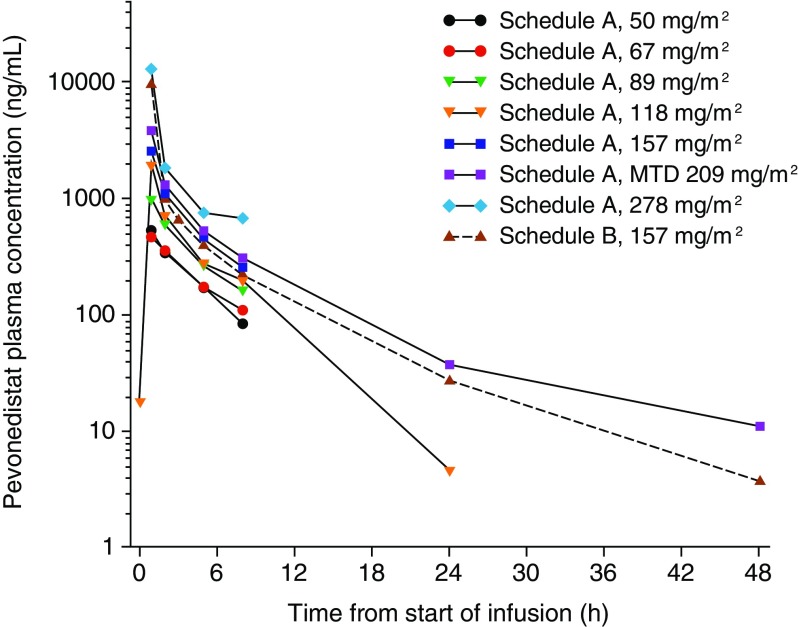
Mean pevonedistat plasma concentration–time profiles on Cycle 1, Day 1 following 1-h intravenous infusion of pevonedistat in patients receiving different dose levels on schedule A (Days 1, 4, 8, and 11) and patients receiving the one dose level on schedule B (Days 1, 8, and 15)

A total of 34 patients were evaluable for PK, including 24 patients on schedule A (2 patients each treated at pevonedistat 50 and 67 mg/m^2^, 1 at 89 mg/m^2^, 5 at 118 mg/m^2^, 1 at 157 mg/m^2^, 11 at 209 mg/m^2^, and 2 at 278 mg/m^2^) and 10 on schedule B. Mean pevonedistat plasma concentration–time profiles on Cycle 1, Day 1 for all patients are shown in Fig. 1. On schedule A, the majority of individual PK profiles were truncated at the 7-h post-infusion time point due to missing subsequent samples. Therefore, pevonedistat systemic exposure (as assessed by area under the plasma concentration–time curve [AUC]) could not be accurately estimated, except at the MTD. Based on limited data availability in both schedules, mean plasma exposure of pevonedistat (maximum plasma concentration or AUC when available) increased approximately proportionally with dose from 50 to 278 mg/m^2^ after Day 1 intravenous infusion. Individual PK profiles across schedules A and B showed a biphasic disposition phase following completion of the intravenous infusion. Plasma concentrations were generally quantifiable between 24 and 72 h after dosing across the dose range studied. Pevonedistat PK parameters at the schedule A MTD of 209 mg/m^2^ and at 157 mg/m^2^ on schedule B are summarized in Table 3.

**Table 3:** Pharmacokinetic (PK) parameters of pevonedistat on Cycle 1, Day 1 in evaluable patients administered pevonedistat via a 1-h infusion on schedule A (Days 1, 4, 8, and 11) and on schedule B (Days 1, 8, and 15)

Parameter*	Schedule A MTD, 209 mg/m^2^ (*n* = 11)	Schedule B, 157 mg/m^2^ (*n* = 10)
C_max_, ng/mL	3591 (41)	2452 (17)**
T_max_, h^†^	1.00 (0.98–1.2)	1.00 (0.92–1.7)**
AUC_24hr_, ng.h/mL	10,716 (13)^#^	7248 (24)^#^
AUC_inf_, ng.h/mL	12,300^§^	8932 (19)^‡‡^
t½, h^‡^	10.4^§^	10.1 (0.92)^¶^
CL_p_, L/h	38.1^§^	38.8 (16)^##^
V_ss_, L	237^§^	245 (10)^##^

*Geometric mean (% coefficient of variance) except where indicated. ^†^Median (range). ^‡^Arithmetic mean (standard deviation). ^#^ *n* = 8. ^¶^ *n* = 5. ^§^ *n* = 1. **n = 9. ^‡‡^ *n* = 4. ^##^ *n* = 3

*AUC*_*24hr*_ */AUC*_*inf*_ area under the plasma concentration–time curve from time 0 to 24 h / extrapolated to infinity, *CL*_*p*_ systemic (plasma) clearance, *C*_*max*_ observed maximum plasma concentration, *T*_*max*_ time to C_max_, *t½* terminal disposition phase half-life, *V*_*ss*_ volume of distribution at steady state

### Pharmacodynamics

A panel of NAE-regulated transcripts was measured (by RT-PCR) in whole blood samples collected on study. All eight NAE-regulated gene transcripts were significantly increased post-treatment among patients receiving pevonedistat at the MTD on schedule A and patients receiving pevonedistat on schedule B (Supplementary Fig. S2, Supplementary Table S1). Induction of all NAE-regulated transcriptional targets was observed at pevonedistat doses of 50 mg/m^2^ and higher (data not shown). On schedule A, changes were characterized by rapid increases in gene transcript levels that remained increased compared with baseline during the first 7 h following pevonedistat dosing on Days 1 and 11, when assessments were conducted. Changes were heterogeneous between patients at a given dose level and between gene transcripts at a given dose level, as shown for the pevonedistat MTD of 209 mg/m^2^ in Supplementary Figure S2. SLC7A11 and NQ01 were consistently the most robustly increased gene transcripts. Findings were similar on schedule B (Supplementary Fig. S2).

**Fig. 2:**
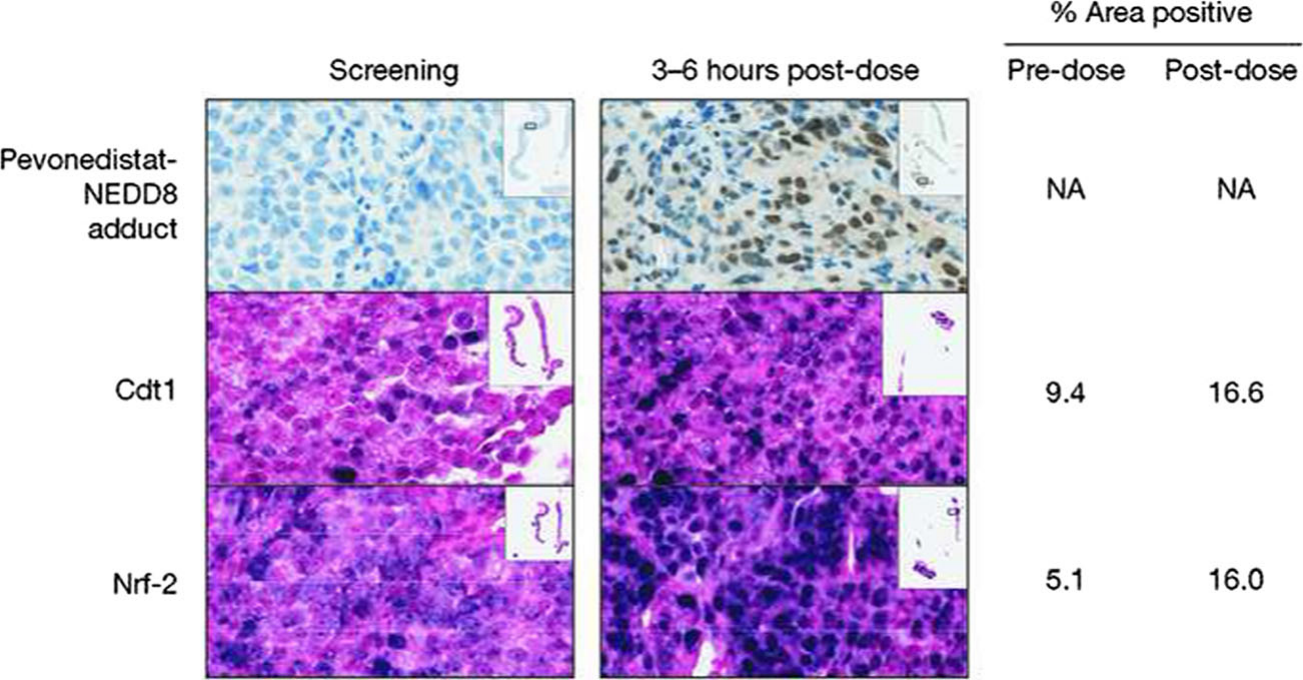
Representative immunohistochemistry images of tumor formalin-fixed paraffin-embedded serial sections stained for pevonedistat–NEDD8 adduct, Cdt1, and Nrf-2, at screening/baseline and at 3–6 h after the Day 4, Cycle 1 dose, indicating increases in post-dose staining for all three markers (patient received pevonedistat 209 mg/m^2^ on schedule A)

Tumor biopsies were collected at screening and at 3–6 h after the Cycle 1, Day 4 dose of pevonedistat from 3 patients in the Schedule A MTD expansion cohort. All of the Day 4 post-dose biopsy samples were positive for the pevonedistat–NEDD8 adduct, demonstrating penetration of pevonedistat into the tumors. Additionally, in one patient with adequate biopsy tissue, it was feasible to analyze the immunohistochemical expression of Cdt1 and Nrf-2, both of which are substrates of CRLs; expression of both proteins was increased post-treatment (Fig. 2).

### Clinical activity

**Fig. 3:**
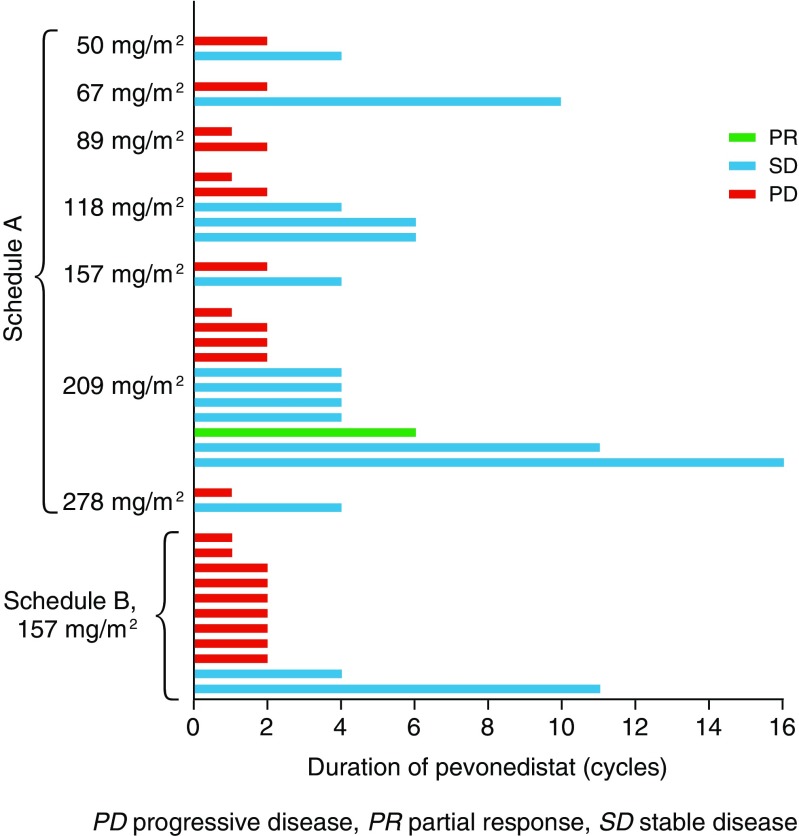
Duration of pevonedistat exposure among all 37 patients on schedules A and B, including the 15 patients achieving stable disease and the 1 patient achieving a partial response

Thirty-one of 37 patients were evaluable for response, including 23 in schedule A and 8 in schedule B. Figure 3 indicates the duration of pevonedistat exposure among all 37 patients across both schedules, and highlights the patients who achieved best responses of partial response or stable disease. These included one (3 %) partial response, achieved by a patient on schedule A who received pevonedistat at the MTD (209 mg/m^2^). Another 15 (48 %) patients on schedule A and B had a best response of stable disease; the remaining 15 (48 %) patients had progressive disease.

The patient who achieved a partial response was a 61-year-old white female with stage IV malignant melanoma (tumor sites in the brain, lung, and skin). She had received prior antineoplastic therapy (tremelimumab), radiation therapy, and surgery, and received 6 cycles of pevonedistat 209 mg/m^2^. The partial response was reported at Cycle 4 (Supplementary Fig. S3 includes PET scans showing tumor reduction) and was maintained until an assessment of progressive disease after Cycle 6, which resulted in discontinuation of study treatment. The duration of response was 1.55 months, and the overall duration of stable disease or better was 4.4 months. Among the 15 patients who achieved stable disease, 4 had stable disease for 6.5 months or longer (Fig. 3); none of these 4 patients had a *BRAFV600E* mutation.

## Discussion

This phase I study investigated the safety of two different administration schedules of pevonedistat, a first-in-class NAE inhibitor, in patients with metastatic melanoma. The study also provided useful insights into the efficacy, PK, and PD profile of pevonedistat in these patients. NAE inhibition with pevonedistat resulted in the anticipated PD effects in the tumor microenvironment in patients with metastatic melanoma. These findings, along with data from other phase I studies of pevonedistat in other solid tumors and hematologic malignancies [29, 33, 35], have helped inform the clinical development of this agent, which is ongoing in combination with azacitidine in patients with acute myeloid leukemia (AML) (NCT01814826) [36] and in combination with docetaxel, gemcitabine, and carboplatin–paclitaxel in patients with solid tumors (NCT01862328). A randomized phase II study of pevonedistat plus azacitidine versus single-agent azacitidine in patients with higher risk myelodysplastic syndromes (MDS), chronic myelomonocytic leukemia, and low blast AML is currently recruiting patients (NCT02610777).

The pevonedistat MTD achieved in the present study on schedule A (Days 1, 4, 8, and 11 of 21-day cycles), of 209 mg/m^2^, appears similar to the MTD of 196 mg/m^2^ established using the same dosing schedule in a study in patients with multiple myeloma or lymphoma [33]. This contrasts with the lower MTDs seen with slightly more dose-intensive schedules in other trials. The MTDs were 110 mg/m^2^ using a Day 1, 2, 8, and 9 schedule in patients with multiple myeloma or lymphoma [33] and 50–67 mg/m^2^ using Days 1–5 and Days 1, 3, and 5 schedules in a study in patients with solid tumors [29]. Of particular note, the MTD of single-agent pevonedistat was also lower when administered on Days 1, 3, and 5 of 21-day cycles in patients with relapsed/refractory AML or MDS (50/59 mg/m^2^; compared with 83 mg/m^2^ using a Days 1, 4, 8, and 11 schedule in the same study) [35, 37]. The MTD was even lower (20 mg/m^2^) when pevonedistat was investigated on the same schedule in combination with azacitidine in treatment-naïve elderly patients with AML [36].

The reasons for this substantial range in the established MTDs are not clear, although the lower MTDs were reported in studies employing more frequent dosing, predominantly on Days 1, 3, and 5. It is plausible that dosing every other day may not have allowed sufficient organ recovery time between doses. This is supported by the safety profile on schedule B (weekly dosing) in the present study; a dose of 157 mg/m^2^ resulted in only 1 DLT in 11 patients. However, the MTDs may also have been influenced by individual patient- and disease-specific characteristics and their association with the key pevonedistat-related toxicities. Thus, comparisons between different studies in different patient populations should be made with caution. Of note, ongoing studies of pevonedistat in combination are employing a Days 1, 3, and 5 dosing schedule [36].

The DLTs and other key toxicities observed on schedule A in the present study, which included hypophosphatemia, acute renal and hepatic toxicity, and acute respiratory failure, appear similar to those reported in other phase I studies. These toxicities were usually observed soon after the first dose and did not occur in patients who tolerated the first few doses well. In the phase I study of pevonedistat in multiple myeloma and lymphoma, DLTs included febrile neutropenia, AST elevation, muscle cramps, and thrombocytopenia [33]. The phase I study of pevonedistat in solid tumors reported DLTs of ALT elevation, AST elevation, and hyperbilirubinemia [29]. In the AML/MDS phase I study, multi-organ failure, reversible ALT elevation, AST/ALT elevation, cardiac failure, and acute renal failure were among the reported DLTs [35, 37].

Most patients tolerated pevonedistat well. The precise reasons for the occurrence of acute severe toxicities at higher pevonedistat doses in a few patients is unclear at this time. Preclinical studies of mechanism-based toxicity with pevonedistat showed increased levels of markers of tissue injury, such as ALT/AST, bilirubin, and creatinine, increased circulating cytokines, and organ damage in rodents receiving pevonedistat combined with tumor necrosis factor-alpha [38]. This suggests a mechanism potentially giving rise to the acute organ failure toxicities reported in the present study and other phase I studies. Based on a program-wide safety review, doses of pevonedistat at the higher end of the range studied (>100 mg/m^2^) are not being considered for further investigation.

The limited PK data from this study indicate that plasma exposure of pevonedistat following completion of the intravenous infusion was approximately dose-proportional across the 50–278 mg/m^2^ dose range. Pevonedistat showed a biphasic disposition profile characterized by a short elimination half-life of approximately 10 h in plasma. These findings are consistent with the PK profile of pevonedistat obtained from other phase I studies in different tumor types using similar or different dosing schedules [29, 33, 35, 37].

PD studies demonstrated the anticipated effects of NAE inhibition at all doses tested in this study. For example, the RT-PCR data showing increases in NAE-regulated gene transcripts and the tumor biopsy stains for the CRL substrates Cdt1 and Nrf-2 are supportive of the mechanism of action of NAE inhibition and subsequent CRL inactivation. Increases in these CRL substrates have also been seen in preclinical studies of pevonedistat [7, 13, 16, 19, 21, 22], and similar PD effects have been reported from other clinical studies [29, 33, 35–37]. Measurement of NAE-regulated gene transcript levels provides a simple, indirect way of observing the anticipated build-up of CRL target proteins as a result of NAE inhibition. Our findings further validate the use of NAE-regulated transcriptional targets as PD markers of NAE inhibition in the clinic. The PD studies also confirmed that pevonedistat was reaching its intended target; immunohistochemical stains of tumor biopsies indicated that the drug penetrated into the tumor tissue and formed the anticipated pevonedistat–NEDD8 adduct in the presence of activated NAE [8].

The relevance of the efficacy results of this study to the current therapeutic landscape of metastatic melanoma is unclear. At the time of study initiation, there was a substantial unmet need for novel treatment approaches in this setting, and the rationale for the investigation of pevonedistat in metastatic melanoma was supported by preclinical studies showing the relevance of the mechanism of action, as evidenced by cytotoxicity and antitumor activity [24–28]. In the present study, a partial response was reported in one patient and stable disease was reported in 15 patients (lasting for 6.5 months or more in 4 patients). Pevonedistat monotherapy currently has limited utility in the context of the broader melanoma treatment landscape, which has evolved rapidly due to the recent emergence of a number of new therapies, offering improved outcomes [39]. These new drugs, including ipilimumab (an immune checkpoint inhibitor targeting CTLA-4) [40], pembrolizumab and nivolumab (monoclonal antibodies that inhibit the PD-1 receptor) [41, 42], vemurafenib [43] and dabrafenib [44] (BRAF inhibitors), and trametinib (MEK inhibitor) [45], have resulted in substantial efficacy and improvements in outcomes. Pevonedistat, with its good safety profile in most patients and clinically meaningful antitumor activity in some patients, may be useful as a combination partner for use with other melanoma therapies. Interestingly, all of the patients with a partial response or durable stable disease had wild-type *BRAF* melanoma. This subgroup of melanoma has a clear unmet need for effective molecularly targeted therapies and could benefit from further investigation of pevonedistat in combination with other therapies.

In conclusion, this study has provided further evidence, in addition to phase I studies in other solid tumors and hematologic malignancies, that NAE inhibition with pevonedistat results in the anticipated PD effects and that pevonedistat reaches the tumor target in metastatic melanoma patients. The data from this study have contributed to the characterization of the safety profile of this first-in-class agent, studies of which are currently ongoing in AML in combination with azacitidine [36] and in solid tumors in multiple combinations.

## Electronic supplementary material

Below is the link to the electronic supplementary material.

Supplementary Table S1 (DOC 32 kb)

Supplementary Table S2 (DOC 51 kb)

Supplementary Fig. S1 Dose-escalation schema, using a Bayesian continual reassessment method. CRM, continual reassessment method; DLT, dose-limiting toxicity; Mid_high_, midpoint between current dose level and next dose level; Mid_low_, midpoint between previous dose level and current dose level; MTD, maximum tolerated dose; PMTD, predicted maximum tolerated dose. Starting dose presented (50 mg/m^2^) was for schedule A. Starting dose for schedule B was 157 mg/m^2^. ^a^Dosing intervals were to be 1.33-fold over the previous dose level and were not determined by the CRM algorithm. (GIF 24 kb)

High Resolution Image (EPS 1137 kb)

Supplementary Fig. S2 Percent change from baseline over time of NAE-regulated gene transcripts in whole blood during Cycle 1 of pevonedistat dosing at the MTD of 209 mg/m^2^ on schedule A or at 157 mg/m^2^ on schedule B. Increases in gene transcript levels can be seen across the genes after pevonedistat dosing on days 1 and 11 in schedule A and days 1 and 15 in schedule B. Dotted lines represent individual patients; symbols represent data obtained at specified times. MTD, maximum tolerated dose. (GIF 116 kb) (GIF 132 kb)

High Resolution Image (EPS 1380 kb) High Resolution Image (EPS 1771 kb)

Supplementary Fig. S3 Tumor reduction in a 61-year-old woman with malignant melanoma who progressed through multiple (>6) prior therapies before receiving pevonedistat 209 mg/m^2^ on schedule A, achieving a partial response after 4 treatment cycles prior to an assessment of progressive disease in Cycle 6. (GIF 58 kb)

High Resolution Image (TIF 1507 kb)
