# Supplementary material for: A phase I study of the investigational NEDD8-activating enzyme inhibitor pevonedistat (TAK-924/MLN4924) in patients with metastatic melanoma
Source: Invest New Drugs. 2016 Apr 8;34:439–49. doi: 10.1007/s10637-016-0348-5 (PMC4919369; doi:10.1007/s10637-016-0348-5)
Supplement: Supplementary file 1 — (DOC 32 kb) [file 10637_2016_348_MOESM1_ESM.doc]

**Supplementary Table S1** Percentage change from baseline of expression levels of transcripts of NAE-regulated genes by RT-PCR (reverse transcription polymerase chain reaction) following Cycle 1, Day 1 pevonedistat dosing at the MTD of 209 mg/m^2^ on schedule A (percentage change at 7 h post-dose) or at 157 mg/m^2^ on schedule B (percentage change at 8 h post-dose)

| **Transcriptional target** | **Schedule A MTD, 209 mg/m^2^ (*n*=11);**  **Median % change (range)** | **Schedule B, 157 mg/m^2^ (*n*=8);**  **Median % change (range)** |
| --- | --- | --- |
| ATF3 | 88.6 (–38.6 to 385.0) | 260 (30.8 to 541.4) |
| GCLM | 105.3 (–33.0 to 234.2) | 65.4 (20.0–108.0) |
| GSR | 196.6 (–54.2 to 447.6) | 176.2 (93.2–216.6) |
| MAG1 | 99.1 (–22.4 to 236.4) | 81.7 ( 39.0–158.2) |
| NQ01 | 681.2 (–49.7 to 2740.6) | 833.8 (330.6–2202.3) |
| SLC7A11 | 837.0 (–65.3 to 3873.9) | 727.4 (184.7 to 1660.0) |
| SRXN1 | 142.0 (–50.5 to 340.0) | 81.5 (43.3 to 185.3) |
| TXNRD1 | 220.7 (–41.7 to 347.7) | 156.9 (87.8 to 234.9) |

ATF3, activating transcription factor 3; GCLM, glutamate-cysteine ligase, modifier subunit; GSR, glutathione reductase; MAG1, 1-acylglycerol-3-phosphate O-acyltransferase 9; MTD, maximum tolerated dose; NQ01, NAD(P)H dehydrogenase, quinone 1; SLC7A11, solute carrier family 7, member 11; SRXN1, sulfiredoxin 1; TXNRD1, thioredoxin reductase 1.
