# Supplementary material for: A phase I study of the investigational NEDD8-activating enzyme inhibitor pevonedistat (TAK-924/MLN4924) in patients with metastatic melanoma
Source: Invest New Drugs. 2016 Apr 8;34:439–49. doi: 10.1007/s10637-016-0348-5 (PMC4919369; doi:10.1007/s10637-016-0348-5)
Supplement: Supplementary file 2 — (DOC 51 kb) [file 10637_2016_348_MOESM2_ESM.doc]

**Supplementary Table S2** Most common treatment-emergent AEs reported in at least 15% of patients overall, and all treatment-emergent grade ≥3 AEs

| **AE, *n* (%)** | **Schedule A (*n*=26)** | **Schedule B (*n*=11)** | **Total (*N*=37)** |
| --- | --- | --- | --- |
| Common AEs (≥15% of patients): |  |  |  |
| Fatigue | 18 (69) | 7 (64) | 25 (68) |
| Diarrhea | 11 (42) | 7 (64) | 18 (49) |
| Anemia | 10 (38) | 5 (45) | 15 (41) |
| Myalgia | 10 (38) | 5 (45) | 15 (41) |
| Nausea | 10 (38) | 3 (27) | 13 (35) |
| Constipation | 7 (27) | 5 (45) | 12 (32) |
| Vomiting | 9 (35) | 3 (27) | 12 (32) |
| Arthralgia | 7 (27) | 4 (36) | 11 (30) |
| Decreased appetite | 8 (31) | 3 (27) | 11 (30) |
| Dizziness | 7 (27) | 3 (27) | 10 (27) |
| Peripheral neuropathy | 7 (27) | 3 (27) | 10 (27) |
| Back pain | 7 (27) | 1 (9) | 8 (22) |
| Blood ALP increased | 6 (23) | 2 (18) | 8 (22) |
| GGT increased | 6 (23) | 1 (9) | 7 (19) |
| Headache | 7 (27) | 0 | 7 (19) |
| Muscle spasms | 7 (27) | 0 | 7 (19) |
| Pyrexia | 4 (15) | 3 (27) | 7 (19) |
| Anxiety | 4 (15) | 2 (18) | 6 (16) |
| AST increased | 5 (19) | 1 (9) | 6 (16) |
| Chills | 5 (19) | 1 (9) | 6 (16) |
| Cough | 6 (23) | 0 | 6 (16) |
| Dyspnea | 3 (12) | 3 (27) | 6 (16) |
| Hypoalbuminemia | 5 (19) | 1 (9) | 6 (16) |
| Pain | 4 (15) | 2 (18) | 6 (16) |
| Upper respiratory tract infection | 5 (19) | 1 (9) | 6 (16) |
| Grade ≥3 AEs (>1 patient)*: |  |  |  |
| Anemia | 3 (12) | 2 (18) | 5 (14) |
| Small intestinal obstruction | 1 (4) | 1 (9) | 2 (5) |
| Neoplasms benign, malignant and  unspecified (including cysts and polyps) | 1 (4) | 1 (9) | 2 (5) |

*The following grade ≥3 AEs were reported in 1 patient each: abdominal pain, abdominal pain lower, acute hepatic failure, acute renal failure, acute respiratory failure, angina pectoris, anuria, arthralgia, bacteremia, blood bilirubin increased, blood creatinine increased, confusional state, dyspnea exertional, failure to thrive, fatigue, gastrointestinal hemorrhage, GGT increased, hepatic encephalopathy, hyponatremia, hypophosphatemia, hypotension, injury/poisoning/procedural complications, insomnia, intussusception, malignant melanoma, musculoskeletal chest pain, myocarditis, nausea, neoplasm malignant, pain, pain in extremity, pneumonia, respiratory distress, respiratory failure, spinal compression fracture, and syncope.

ALP, alkaline phosphatase; ALT, alanine aminotransferase; AST, aspartate aminotransferase; GGT, gamma-glutamyltransferase
