# Supplementary figures and images for: A phase I study of the investigational NEDD8-activating enzyme inhibitor pevonedistat (TAK-924/MLN4924) in patients with metastatic melanoma
Source: Invest New Drugs. 2016 Apr 8;34:439–49. doi: 10.1007/s10637-016-0348-5 (PMC4919369; doi:10.1007/s10637-016-0348-5)

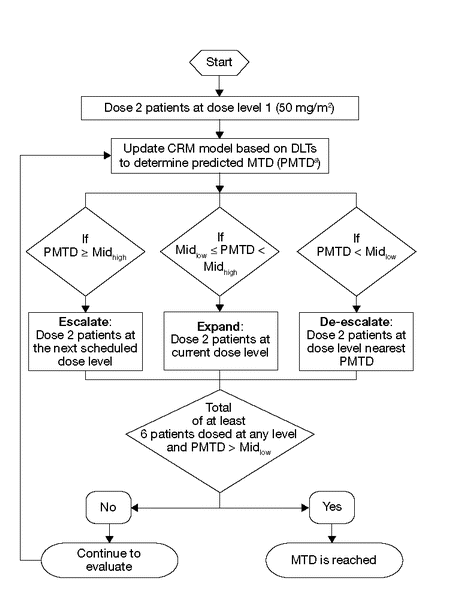

Supplement: Supplementary file 3 — Dose-escalation schema, using a Bayesian continual reassessment method. CRM, continual reassessment method; DLT, dose-limiting toxicity; Midhigh, midpoint between current dose level and next dose level; Midlow, midpoint between previous dose level and current dose level; MTD, maximum tolerated dose; PMTD, predicted maximum tolerated dose. Starting dose presented (50 mg/m2) was for schedule A. Starting dose for schedule B was 157 mg/m2. aDosing intervals were to be 1.33-fold over the previous dose level and were not determined by the CRM algorithm. (GIF 24 kb) [file 10637_2016_348_Fig4_ESM.gif]

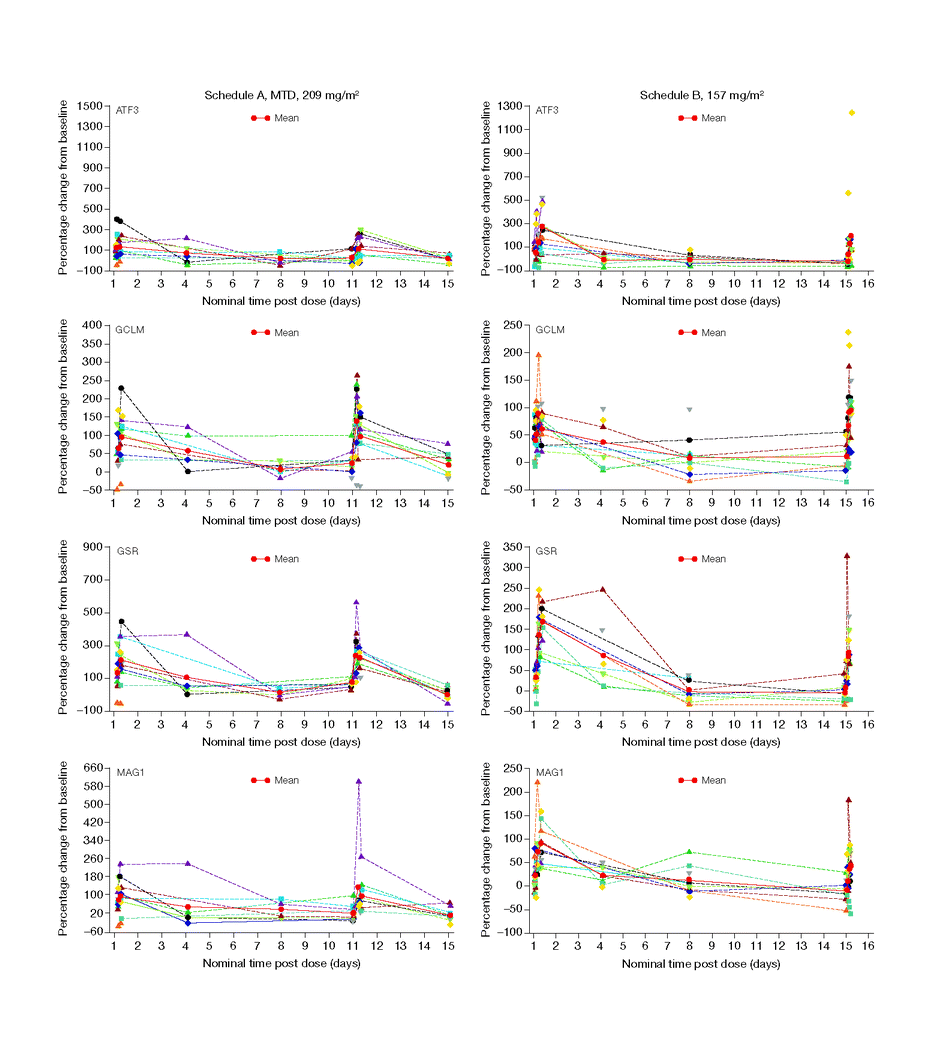

Supplement: Supplementary Fig. S2 — Percent change from baseline over time of NAE-regulated gene transcripts in whole blood during Cycle 1 of pevonedistat dosing at the MTD of 209 mg/m2 on schedule A or at 157 mg/m2 on schedule B. Increases in gene transcript levels can be seen across the genes after pevonedistat dosing on days 1 and 11 in schedule A and days 1 and 15 in schedule B. Dotted lines represent individual patients; symbols represent data obtained at specified times. MTD, maximum tolerated dose. (GIF 116 kb) [file 10637_2016_348_Fig5_ESM.gif]

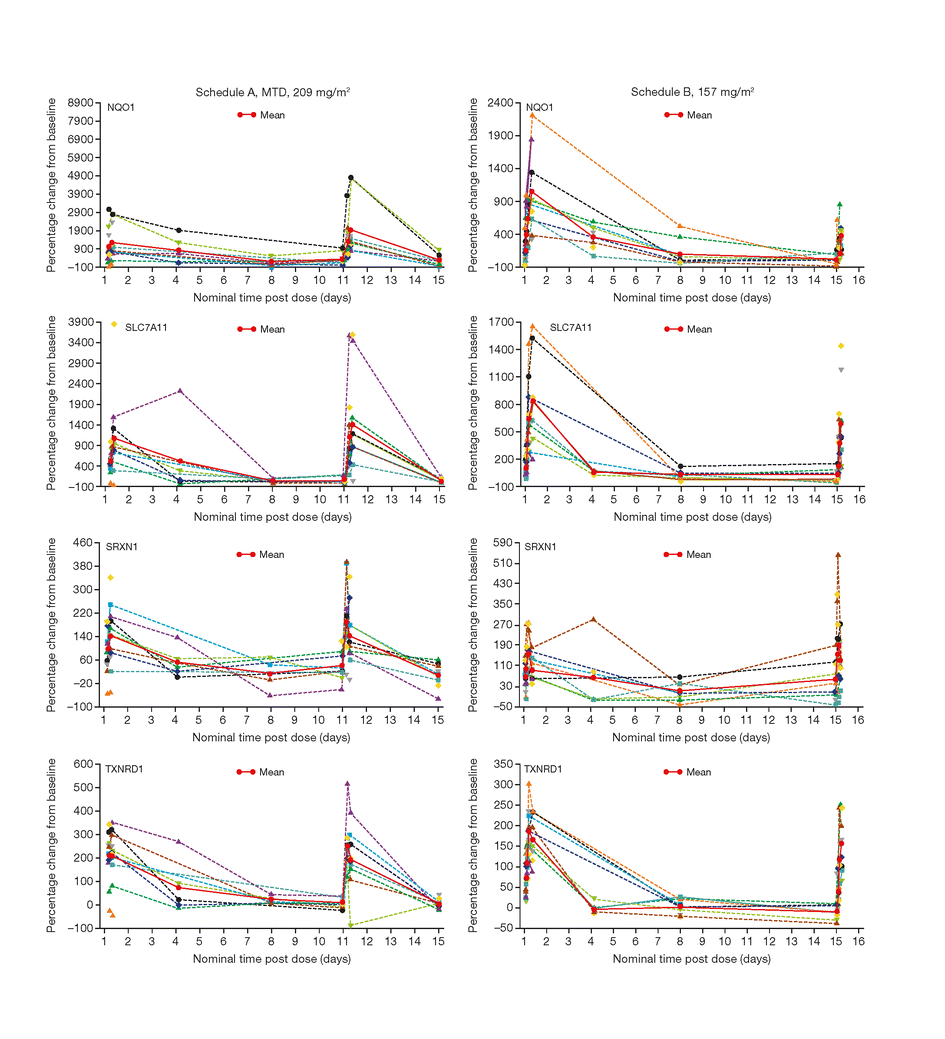

Supplement: Supplementary Fig. S2 — Percent change from baseline over time of NAE-regulated gene transcripts in whole blood during Cycle 1 of pevonedistat dosing at the MTD of 209 mg/m2 on schedule A or at 157 mg/m2 on schedule B. Increases in gene transcript levels can be seen across the genes after pevonedistat dosing on days 1 and 11 in schedule A and days 1 and 15 in schedule B. Dotted lines represent individual patients; symbols represent data obtained at specified times. MTD, maximum tolerated dose. (GIF 116 kb) [file 10637_2016_348_Fig6_ESM.gif]

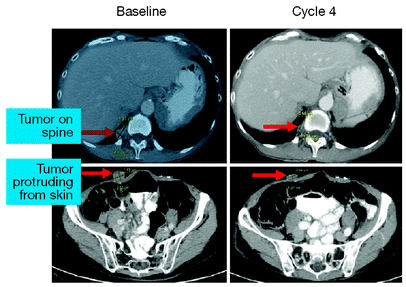

Supplement: Supplementary file 9 — Tumor reduction in a 61-year-old woman with malignant melanoma who progressed through multiple (>6) prior therapies before receiving pevonedistat 209 mg/m2 on schedule A, achieving a partial response after 4 treatment cycles prior to an assessment of progressive disease in Cycle 6. (GIF 58 kb) [file 10637_2016_348_Fig7_ESM.gif]

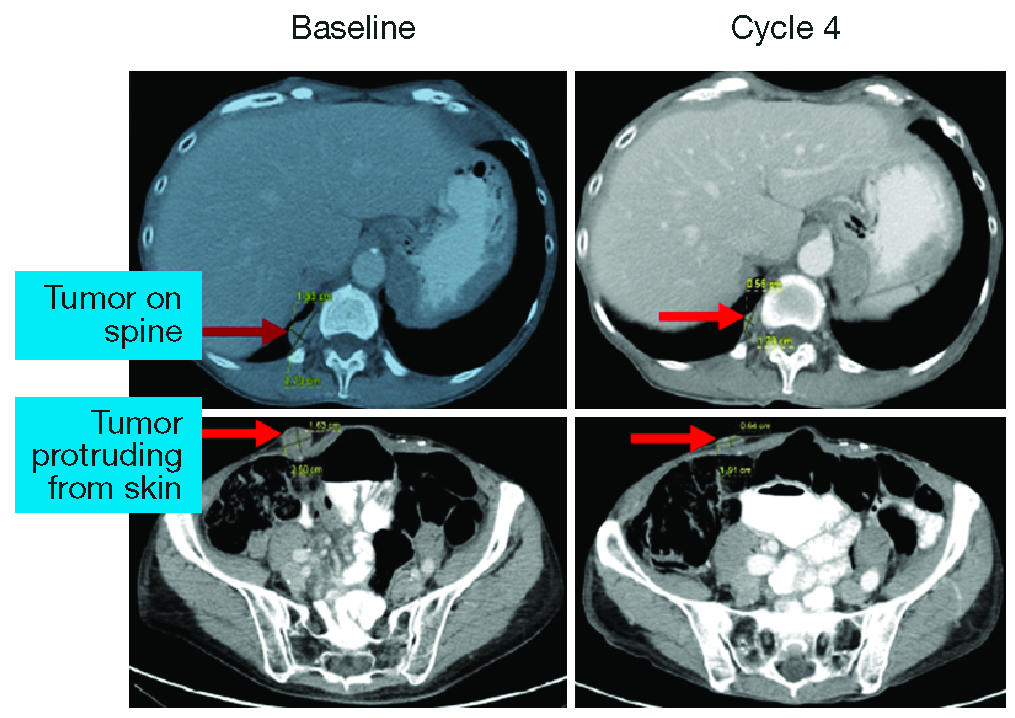

Supplement: Supplementary file 10 — High Resolution Image (TIF 1507 kb) [file 10637_2016_348_MOESM6_ESM.tif]
